# Gut microbiota of sub-Saharan Africa infants exposed to antiretroviral therapy: Scoping review

**DOI:** 10.4102/phcfm.v17i1.4939

**Published:** 2025-09-29

**Authors:** Taona E. Mudhluli, Runyararo Mashingaidze-Mano, Inam Chitsike, Justen Manasa, Lindsay J. Hall, Exnevia Gomo, Danai T. Zhou

**Affiliations:** 1 Department of Biochemistry, Faculty of Science, Midlands State University, Gweru, Zimbabwe; 2 Department of Laboratory Investigative and Diagnostic Sciences, Faculty of Medicine and Health Sciences, University of Zimbabwe, Harare, Zimbabwe; 3 School of Medicine, Faculty of Health Sciences and Veterinary Medicine, University of Namibia, Windhoek, Namibia; 4 Department of Maternal and Child Health, Faculty of Medicine and Health Sciences, University of Zimbabwe, Harare, Zimbabwe; 5 Department of Microbes, Infection and Microbiomes, College of Medicine and Health, University of Birmingham, Birmingham, United Kingdom

**Keywords:** antiretroviral drugs, gut microbiota, human immunodeficiency virus, infants, sub-Saharan Africa

## Abstract

**Background:**

Antiretroviral (ARV) exposure influences the early-life gut microbiota in regions with high human immunodeficiency virus (HIV) burdens. Understanding how ARV drugs affect the infant gut microbiota is important for optimising short-term and long-term health outcomes.

**Aim:**

This scoping review synthesises current evidence on the gut microbiota of infants born to mothers with HIV (MWH) in sub-Saharan Africa, focusing on the effects of in utero and postnatal ARV exposure. By examining emerging data in this context, we highlight potential implications for infant health and identify key areas for future research.

**Method:**

Online databases were systematically searched using comprehensive search strategies. In addition, grey literature was explored. Three authors independently screened titles and abstracts for relevance, evaluated full-text articles for eligibility and performed data extraction.

**Results:**

The scoping review highlights differences in gut microbiota because of HIV exposure and ARV drugs in infants born to sub-Saharan African MWH. Of interest is a disturbance in the gut bacterial balance in infants with HIV, who harboured enriched with more diverse and potentially harmful bacteria relative to HIV-exposed uninfected infants. There was agreement from some countries, that is Nigeria and Zimbabwe, that their gut microbiota genomes comprise *Bifidobacterium longum* subspecies *infantis* and *Enterococcus*.

**Conclusion:**

Both antiretroviral therapy and HIV influence the gut microbiota in infants born to MWH. Pathogenic overgrowth within the infant gut microbiota for individuals with HIV may impair immune maturation during early-life, with lasting consequences for host health.

**Contribution:**

This highlights the need for further research into probiotic interventions for infants in high HIV-burden settings.

## Introduction

In the sub-Saharan African region, the gut microbiota of more than 1.5 million infants is exposed to antiretroviral (ARV) drugs because of the high prevalence of human immunodeficiency virus (HIV).^1,2^ The use of ARV drugs has been a game-changer in preventing mother-to-child transmission of HIV infection (PMTCT) since the early 2000s.^1,2^ This strategy has significantly reduced the risk of HIV transmission from mother-to-child during pregnancy, childbirth and breastfeeding.^3^ The ground-breaking Paediatric AIDS Clinical Trial Group (PACTG) Study 076, conducted in 1994, marked a significant milestone in PMTCT.^3^ This pivotal study demonstrated the efficacy of zidovudine (ZDV) monotherapy in reducing the risk of HIV transmission from mother to child.^3^ Building on this success, subsequent trials explored more comprehensive regimens, combining ZDV with other ARV agents such as lamivudine (3TC), nevirapine (NVP) and protease inhibitors (PIs).^3^ These studies investigated various antepartum, intrapartum and postpartum treatment strategies, ultimately shaping the evolution of PMTCT guidelines and paving the way for more effective, multi-drug approaches to prevent HIV transmission and improve maternal and infant health outcomes.^3^ The most recent PMTCT programme, the Option B+, initiates all pregnant or breastfeeding women with HIV onto lifelong triple antiretroviral therapy (ART), regardless of their CD4 count or clinical stage, promoting better adherence and health outcomes.^1,2,3^ Since the introduction of PMTCT worldwide, HIV transmission rates from mothers to their children have declined from more than 25% to less than 2%.^3^

### Recommendations by the World Health Organization for infant prophylaxis

According to current World Health Organization (WHO) guidelines, postnatal prophylaxis for infants born to mothers with HIV (MWH) typically involves administering either NVP or ZDV for 4 to 6 weeks.^4^ In cases where breastfeeding infants are at high risk of HIV transmission, the prophylaxis may be extended to 12 weeks.^4^ In the PMTCT context, ARV prophylaxis is the administration of ARV drugs to infants born to MWH.^5^ Despite being used by over 1.5 million infants in sub-Saharan Africa (SSA), the impact of ARVs on the body and the microbial communities within them – the microbiota – remains incompletely understood, highlighting the need for further research.^6^

### Potential effect of different antiretroviral drugs on the human gut microbiota

The potential effect of different ARV drugs on the gut microbiota of HIV-exposed infected (HEI) and HIV-exposed uninfected (HEU) individuals has been described from *in vitro* experiments (Table 1),^7,8^ but largely overlooked in human microbiome studies. It is plausible that either HIV exposure, HIV infection or ARV drugs impact the early microbiota of infants born to MWH at an early age.^1,9,10^ Furthermore, *in utero*-HEU individuals have a higher incidence of hospitalisation and 2 to 4-fold higher mortality rate than HIV-unexposed uninfected (HUU) infants in low- and middle-income countries (LMICs).^11,12,13^ However, there are inconsistencies in the few published reports on infant gut microbiota from SSA. Therefore, this review will synthesise current key findings from SSA on the gut microbiota of HIV-exposed (HE) infants (born to MWH) on prolonged and short-term ART.

**TABLE 1:** Antiretroviral effects of relevant drugs from recent *in vitro* studies.

ARV drug	Abbreviation	Tested bacterial cultures			
		*Escherichia coli*	*Bacteroides* sp.	*Segatella* (formerly *Prevotella)* sp.	*Enterococcus faecalis*
**NNRTI**					
Nevirapine†	NVP	0% susceptible	70% susceptible	100% susceptible	100% susceptible
**NRTI**					
Zidovudine†	ZDV	100% susceptible	75% susceptible	70% susceptible	0% susceptible
Tenofovir†	TFV	0% susceptible	0% susceptible	0% susceptible	0% susceptible
Tenofovir disoproxil† fumarate	TDF	0% susceptible	0% susceptible	0% susceptible	0% susceptible
**INSTI**					
Dolutegravir‡	DTG	0% susceptible	Not applicable	Not applicable	0% susceptible
**Protease Inhibitors** †					
Lopinavir†	LPV	0% susceptible	0% susceptible	0% susceptible	0% susceptible
Ritonavir†	RTV	0% susceptible	0% susceptible	0% susceptible	0% susceptible
**Combinations**					
TDF/FTC/EFV†	-	0% susceptible	Susceptible (level not specified)	Susceptible (level not specified)	Susceptible (level not specified)
3TC/ZDV/EFV†	-	susceptible (level not specified)	Susceptible (level not specified)	Susceptible (level not specified)	Susceptible (level not specified)

*Source:* †, Ray S, Narayanan A, Giske CG, Neogi U, Sönnerborg A, Nowak P. Altered gut microbiome under antiretroviral therapy: Impact of efavirenz and zidovudine. ACS Infect Dis. 2020;7(5):1104–1115. https://doi.org/10.1021/acsinfecdis.0c00536;

‡ , Rubio-Garcia E, Ferrando N, Martin N, et al. In vitro antibacterial activity of antiretroviral drugs on key commensal bacteria from the human microbiota. Front Cell Infect Microbiol. 2024;13:1306430. https://doi.org/10.3389/fcimb.2023.1306430

ARV, antiretroviral; NNRTI, non-nucleoside reverse transcriptase inhibitors; NRTI, nucleoside reverse transcriptase inhibitors; INSTI, integrase strand transfer inhibitor; FTC, emtricitabine; EFV, efavirenz; 3TC, lamivudine.

### Recommendations by the World Health Organization for preventing human immunodeficiency virus mother-to-child transmission

The WHO outlines four key stages of the PMTCT of HIV cascade. These include pre-pregnancy, prenatal, intrapartum and postnatal interventions (Table 2).^14^ These stages are crucial for a comprehensive approach to preventing HIV transmission from mother to child. According to the current WHO guidelines, high-risk infants born to MWH should receive dual prophylaxis with daily ZDV and NVP for the first 6 weeks of life, regardless of whether they are breastfed or formula-fed.^14^ For breastfed infants at high-risk, including those first identified as HE during the postpartum period, prophylaxis should be continued for an additional 6 weeks, using either dual therapy (ZDV and NVP) or NVP alone.^5,14^ High-risk infants are defined as those born to mothers with documented HIV infection who received less than 4 weeks of ART at delivery, viral load > 1000 copies/mL in the 4 weeks before delivery (if viral load is available),^13^ incident HIV infection during pregnancy or breastfeeding or newly diagnosed HIV during the postpartum period, with or without a prior negative test.^14^ These high-risk infants require extended prophylaxis. In addition, breastfeeding infants of mothers on ART typically receive 6 weeks of daily NVP prophylaxis, while formula-fed infants usually receive 4 weeks to 6 weeks of daily NVP or twice-daily ZDV prophylaxis.^14,15^ Since the introduction of the ‘treat all’ approach, ART is initiated urgently among all pregnant and breastfeeding MWH, even if they are identified late in pregnancy or postpartum.^14,15^ For mothers who initiate ART around the time of labour and delivery (intrapartum), earlier WHO guidelines suggested administering a single dose of NVP along with tenofovir disoproxil fumarate (TDF)/emtricitabine (FTC), in conjunction with 3-hourly administration of ZDV during labour.^14,16^

**TABLE 2:** Four main aspects of preventing mother-to-child transmission of human immunodeficiency virus cascade outlined by the World Health Organization.

Maternal HIV status	Antiretroviral drug intervention status for PMTCT			
	Negative low-risk	Negative high-risk	MWH high-risk	MWH low-risk
Before pregnancy	No ART exposure	Adult PrEP containing TDF Adult PEP comprising TDF + 3TC + DTG	No ART exposure *HIV status known at delivery or MWH not taking ART*	First or second-line ART
Prenatal infant exposure	No ART exposure	in utero exposure	No ART exposure	*In utero* ART exposure
Intrapartum infant exposure	No ART exposure	in utero exposure	No ART exposure	*In utero* ART exposure
Postnatal infant exposure	No ART exposure	NVP or ZDV	NVP + ZDV and NVP or ZDV	NVP or ZDV during breastfeeding

*Source*: World Health Organisation. Consolidated guidelines on HIV prevention, testing, treatment, service delivery and monitoring: Recommendations for a public health approach. 2021.

ART, antiretroviral therapy; MWH, mothers with HIV; NVP, nevirapine; PMTCT, prevention of mother-to-child transmission of HIV; PEP, post-exposure prophylaxis; PrEP, pre-exposure prophylaxis; WHO, World Health Organization; ZDV, zidovudine; TDF, tenofovir disoproxil fumarate; 3TC, lamivudine; DTG, dolutegravir; HIV, human immunodeficiency virus; MWH, mothers with HIV; 3TC, lamivudine.

### The gut microbiota of infants exposed to antiretroviral drugs from sub-Saharan Africa

Some studies suggest that prolonged ART may help restore the gut microbiota of children with HIV (CWH) to a composition more similar to that of HUU children.^17^ A study of children and adolescents (6 years to 16 years) in Zimbabwe found that HUU participants had significantly higher species richness in their gut microbiota compared to HEI individuals.^17^ Notably, prolonged ART was associated with increased gut microbiota richness. However, the duration of ART did not impact beta diversity.^17^ The study’s findings suggest that while prolonged ART may have a positive effect on gut microbiota richness, the microbiota of HEI children on ART remains less diverse compared to HUU children.^17^ In contrast, other studies report a decrease in bacterial diversity after long-term ARV exposure.^9,18^ The impact of ARV drugs on the gut microbiota of infants in SSA is a significant area of interest, given the high prevalence of HIV in the region.^2^ Antiretroviral drugs are believed to disrupt the gut microbiota through their broad-spectrum antibacterial activity, which can affect the balance of gut microorganisms.^7,8^ In previous *in vitro* studies, ARV drugs such as NVP, TFV and ZDV exhibited antibacterial effects.^7,8^ The disruption is thought to occur through mechanisms such as deoxyribonucleic acid (DNA) chain termination, inhibition of bacterial polymerases and induction of the ‘SOS’ DNA damage response.^7,8^ The study’s findings highlight that certain ARV drugs can impact the composition of the human microbiota.

### The current first-line treatment regimen for pregnant women with human immunodeficiency virus

The current first-line treatment regimen recommended for pregnant women with HIV is a fixed-dose combination of TDF, 3TC and dolutegravir (DTG)^14,19^ (Table 2). In several LMICs and SSA countries, current guidelines stipulate that all pregnant women previously exposed to ART, such as through PMTCT programmes or previous interruptions in ART, should take DTG-based ARV drug regimens.^14,19^ All newly-born infants born to MWH receive ART, postpartum if they are diagnosed with HIV, while those who are not infected but are exposed to HIV *in utero*, for example, the HEU infants, receive prophylaxis of either ZDV or NVP.^14,15^

### The gut microbiota of human immunodeficiency virus-unexposed, uninfected infants at birth

In HUU infants, gut microbiota colonisation begins at birth or potentially even in utero, and continues to develop and mature over the first 3 years of life.^20^ The development of the early-life microbiome is influenced by various factors, including birth mode, maternal microbiota, diet (breast milk or formula), antibiotic exposure, medications, geographical location and environmental factors.^20^ The maturing microbiome plays a vital role in shaping a child’s immune system.^21^ Perturbations in the microbiome have been linked to various diseases,^21^ including infectious diseases, highlighting the importance of a balanced microbiome in overall health.^21,22^ The intestinal microbiota of neonates is characterised by low diversity and a relative dominance of the phyla^23,24^ *Actinomycetota* (formerly *Actinobacteria*) and *Pseudomonadota* (formerly *Proteobacteria*).^23,24^ The most abundant classes in infant stool are *Bifidobacterium, Lactobacillus* and *Clostridia* in that order.^25,26^ In healthy breastfed infants, the gut microbiota during the first 1000 days of life is typically dominated by ‘infant-type’ bifidobacteria, including: *Bifidobacterium breve, B. bifidum, B. longum* subsp. *infantis, B. longum subsp. longum* and *B. pseudocatenulatum*.^21^

### Infant gut microbiota perturbations in human immunodeficiency virus-exposed infants

During the first 2 years of life, children are vulnerable to HIV exposure through perinatal transmission (in utero and during delivery) and postnatal transmission (through breastfeeding).^23^ Paediatric HIV infection can disrupt the normal balance of gut microbiota, leading to changes in its composition.^23,24^ Research suggests that exposure to HIV in utero can affect the gut microbiota of HEU infants, indicating a possible connection between the mother’s HIV status and the infant’s gut microbiota.^1^ Previous studies found that *Bifidobacterium* species were significantly less abundant in the gut microbiota of HEU infants during the first 6 months of life.^1^ Metabolites of ART drugs in breast milk have been linked to reduced levels of *Bifidobacterium* in infants.^1^ In HEI infants receiving long-term ART, the disruption to the gut microbiota may persist even when the virus is suppressed.^27,28^ Children with HIV tend to have a gut microbiome with lower bacterial richness, reduced diversity and altered bacterial composition compared to children without HIV.^29^ Differences in study findings on specific taxa altered by HIV infection may be because of variations in geographical location, age, route of HIV exposure and methodological approaches (sequencing and bioinformatics).^20^ Despite these differences, research consistently shows an increased abundance of bacteria linked to immune inflammation and decreased abundance of bacteria that support gut health and integrity.^20^ A study of perinatally infected CWH in India found an increased abundance of *Segatella* (formerly *Prevotella*), a bacterial taxon linked to increased microbial translocation (sCD14) and immune inflammation (IP-10).^20^

## Methods

### Review strategy

**FIGURE 1:**
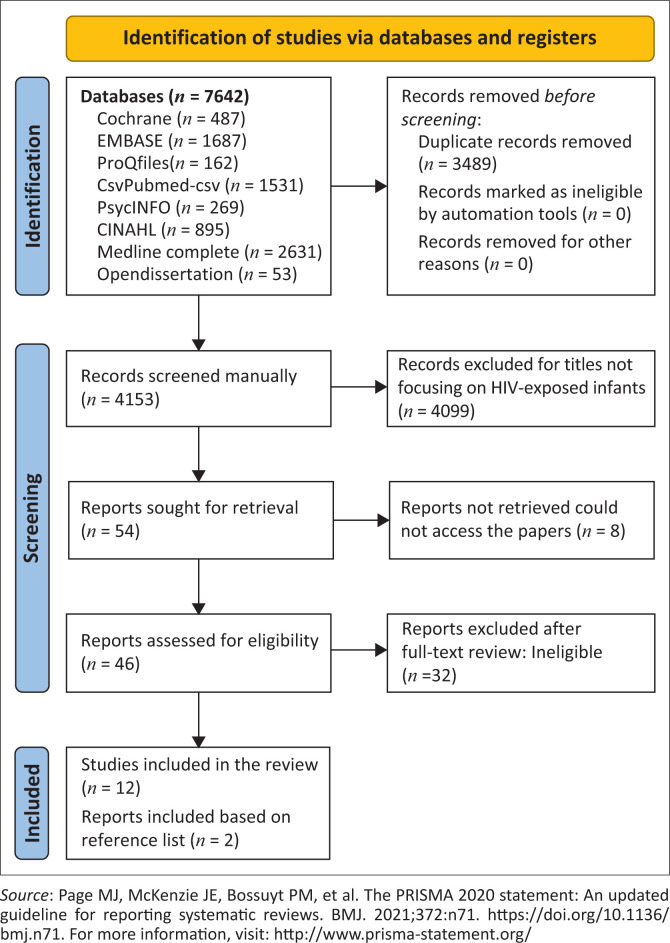
Results from the scoping review search.

Preferred Reporting Items for Systematic Reviews and Meta-Analyses (PRISMA)-2020 updated guidelines for reporting scoping reviews were used to guide the search, select relevant studies and report study findings (Figure 1).^30^ The PRISMA-2020 checklist was used to ensure the inclusion of relevant information in the review. The Population, Intervention, Comparison, Outcome and Context (PICOC) mode – Table 3 was used to develop a research question regarding the association between ART exposure and HIV exposure with the gut microbiota of infants from SSA.

**TABLE 3:** Showing the eligibility criteria as guided by the PICOC(S).

Abbreviation	Full term	Criteria for this scoping review
P	Population	Infants who were either HIV-exposed, infected or HIV-exposed, uninfected or on ART prophylaxis;
I	Intervention	HIV or ART
C	Comparison	Neither HIV nor ART
O	Outcomes	Gut microbiota
C	Context	SSA
S	Study designs	All study designs including non-peer-reviewed literature (grey literature)

ART, antiretroviral therapy; SSA, sub-Saharan Africa; HIV, human immunodeficiency virus; PICOC(s), Population, Intervention, Comparison, Outcome and Context Study.

### Data sources and search strategy

The following databases, CINHAL (Cumulative Index to Nursing and Allied Health Literature), Cochrane Library, PubMed CSV, Embase, MEDLINE, Open Dissertation, PsycINFO, ProQuest and PubMed were systematically searched for relevant articles without any restrictions (Figure 1). The databases were searched from inception until May 2024 using variations and combinations of the following keywords: Africa, ART, HIV, infants, microbio*, NVP, SSA and ZDV. The search strategy is in Appendix 1. Open Dissertations, ProQuest Dissertations and Theses Global were searched to identify grey literature. Lastly, a manual search of the reference lists and citations of relevant publications and reviews was conducted to identify additional studies.

### Eligibility criteria and ethics

#### Inclusion criteria

Studies that meet the PICOC(S) framework (Table 3) were included.

#### Exclusion criteria

Studies were excluded if the:

- The study population were HE at an older age than 36 months.
- Full-text and abstract were both unavailable or only the abstract was available, but did not convey the needed data.
- The studies were narrative, scoping or systematic reviews.

### Study selection and data extraction

The Rayyan Systematic Review Management Software combined and de-duplicated articles retrieved from the searches. Two authors T.E.M. and D.T.Z. independently screened the titles and abstracts to identify articles that meet the inclusion criteria (see search strategy in Appendix 1). T.E.M. obtained full-texts of the articles that passed the first screening. D.T.Z. assessed full-text papers that were identified as potentially relevant for inclusion or exclusion. Discrepancies were resolved through discussion with a third author R.M.-M. and by consensus. Studies that met the inclusion criteria were retained for data extraction. Quality assessment was not conducted in this scoping review.

### Data extraction

… and … independently extracted data from each article retained in the full-text screening using a pretested data extraction form developed by the reviewers. Any uncertainties regarding the inclusion of studies were resolved through discussion with a third author (R.M.-M.) and by consensus. The following data were extracted from each article included: (1) bibliographic information, (2) study design, year of study and location, (3) study aims or objectives, (4) study population, (5) sample size, (6) intervention, (7) comparison group, (8) outcome (diversity of gut microbiota).

### Ethical considerations

This was previously published data and did not require ethical clearance. This article followed all ethical standards for research without direct contact with human or animal subjects.

## Review findings

This scoping review shares findings from multiple contexts across the sub-Saharan African region (Table 4).

**TABLE 4:** Infant gut microbiome studies from sub- Saharan African region.

Method/16s region	Country (year)	Age of infants/HIV status	Gut diversity in infants	Health implication
16S rRNA amplicon sequencing / V4 region	Cameroon^29^ (2021)	87 HEI and 82 HUU children, aged 1 to 19 years (HEI infected perinatally), All HEI on ART and co-trimoxazole prophylaxis.	Lower *Segatella* and *Clostridia* in HEI compared to HUU	Poorer gut health and lower production of SCFAs
Stool cultured using specific growth media antibiotic inhibitors and bacteria identified using biochemical tests	Cameroon^31^ (2019)	3–24 months infants (*N* = 80), 41% HEI, 19% HEU and 40% HUU; 61% were male.	Opportunistic bacteria (*Shigella, Klebsiella, Proteus, Pseudomonas* and *Staphylococcus aureus*) associated with infants with HIV	The immune-compromised state in infants with HIV promotes the proliferation of opportunistic bacteria
Stool cultured using specific growth media and PCR detection of stool DNA on an iQ5 Cycler	Mozambique^32^ (2013)	Apparently healthy infants (*N* = 121), 21% HEU, 79% HUU. Appr. 30 infants per age group (< 2 weeks, 3–6 months, 6–12 months).	*S.aureus*, S. *hominis* and *S.parasanguis* more frequent in infants aged ≤ 14 days old (first lactation) perio d) than in older infants	Findings highlight the impact of specific characteristics of human milk composition on the microbiota in the first weeks of breastfeeding
16S rRNA amplicon sequencing/V3 – V4 regions	Nigeria^1^ (2022)	272 infants i.e. 131 HUU, 141 born to MWH (HEU or HEI), followed up from birth to 18 months	Infants not yet breastfeeding → *Pseudomonas, Enterobacter, Klebsiella, Corynebacterium* Breastfeeding → *Bifidobacterium, Streptococcus* and *Enterobacter*. HEI and HEU had lower bifidobacteria compared to HUU	Lower *Bifidobacterium* in neonates, HEU and HEI reduces support of digestive health, diminishes immune function, producing essential nutrients, preventing chronic diseases, aiding infant development. This difference no longer observed at 9 months, 15 months and 18 months postpartum→ solid foods introduced at around 6 months of age
16S rRNA amplicon sequencing/V4 region	South Africa^11^ (2022)	98 HEU and 88 HUU infants followed up from 6 weeks to 62 weeks. Infants from South Africa and Haiti (numbers not specified)	There were differences in the relative abundances of multiple individual taxa between HEU and HUU infant gut microbiota, but not in alpha or beta diversity	Lower abundance of *Coriobacteriaceae*, Bacteroidetes families, and *Pseudomonadota* phylum in HEU infants at 6 and 62 weeks, respectively increase susceptibility to infections or inflammatory conditions
16S rRNA amplicon sequencing/V3-V4 regions	South Africa^33^ (2024)	Compared infants from South Africa and Nigeria. 82 South African (61 HEU and 21 HUU) and 196 Nigerian (141 HEU and 55 HUU).	Exclusively breastfed Nigerian infants, dominated by *Bifidobacterium longum* subspecies *infantis*, were observed at 15 weeks, but not observed among South African infants. Less pronounced *Pseudomonadota* decrease by age, and an increase of *Clostridia* by age. No significant differences in α-diversity, β-diversity, by HIV exposure status in either country	The country of origin was the most influential factor in the infants’ gut microbiota. Plausible explanations may be the difference in profiles of maternal gut and breastmilk microbiota and human milk oligosaccharides influenced by genetics, ethnicity, diet and body mass index (BMI)
Shotgun metagenome sequencing	South Africa^34^ (2024)	4-week-old neonates (*N* = 69), 34 HEU and 35 HUU compared	Pathobionts, including *Shigella flexneri, Shigella boydii*, and *Klebsiella pneumoniae*, were more enriched in the stools of HEU compared to HUU. *B. breve* was significantly enriched in HUU compared to HEU	Gut microbiota alterations are associated with inflammation and microbial translocation
16S rRNA gene amplicon sequencing V6 region	South Africa^35^ (2020)	20 HUU and 16 HEU children from South Africa compared to 21 HUU and 21 HEU children in Belgium, 33 HUU and 16 HEU children in Canada	No difference in α diversity (Shannon index) across groups. Country had the strongest effect on the microbiome; HIV exposure was significant only in Canada	Cohort-specific differences in demographics, feeding, ARV timing and microbiome; Canadian HEU showed distinct composition and reduced SCFA-producing taxa
16S rRNA amplicon sequencing/V4	South Africa^36^ (2022 poster)	Rectal swab samples were collected from 150 infants at 4 weeks, 12 weeks and 24 weeks of age from 79 HIV infected and 71 HEU infants.	If ART was started later than 14 days of age, the shifts in taxa associated with HIV infection were exacerbated (a decrease in *Bifidobacterium* abundance at 4 weeks, and an increase in *Fusobacterium* and *Enterobacter* abundances at later time points). Disturbances in gut microbiota persisted in HIV infected infants compared to HUU. HIV infection was associated with significant shifts in taxa, including increases in *Bacteroides, Fusobacterium* and *Finegoldia*. HIV status is one driver of variation in the infant microbiome	Persisting shifts in the microbiota profile despite early ART, even among breastfed infants, support the need for complementary interventions to improve outcomes. Benefits associated with breastfeeding in conjunction with early ART in maintaining a more *Bifidobacteria*-rich microbiota profile in infants with HIV
16S rRNA amplicon sequencing/V4	Zimbabwe^17^ (2020)	177 HEI and 103 HUU children	HEI had lower alpha diversity and higher beta diversity compared to HUU. Prolonged ART-treatment (≥10 years) was significantly associated with a richer gut microbiota by alpha diversity	Results suggest that prolonged ART may minimise differences in gut microbiota between HEI and HUU
16S rRNA amplicon sequencing/V3-V4	Zimbabwe^37^ (2024)	78 infants, aged 6 weeks 35.9% were HIV-exposed in utero and during breastfeeding. 84.6% exclusively breastfed.	The gut microbiota comprised predominantly of *Bifidobaterium, Streptococcus, Bacteroides, Collinsella* and *Parabacteroides*	No differences in infant stool indices by infant exposure to maternal HIV. The gut microbiota composition did not differ between HEU and HUU
WGS of cultured isolates	Zimbabwe^38^ (2024)	20 stool specimens from 6-week-old infants, HIV status not stated, in a study exploring *Bifidobacterium* and *Enterococcus* isolated from stool	Detected genomes representative of *Bifidobacterium longum* subsp. *infantis, B. longum* subsp. *longum, B. breve, B. pseudocatenulatum, Enterococcus faecium* and *Enterococcus faecalis*	This study identified promising probiotic strains within Zimbabwean isolates, offering the potential for early-life diet and microbial therapies

Note: Please see full reference list of this article: Mudhluli TE, Mashingaidze-Mano R, Chitsike I, et al. Gut microbiota of sub-Saharan Africa infants exposed to antiretroviral therapy: Scoping review. Afr J Prm Health Care Fam Med. 2025;17(1), a4939. https://doi.org/10.4102/phcfm.v17i1.4939 for more information.

ART, antiretroviral therapy; ARV, antiretroviral; *B. breve, Bifidobacterium breve*; *B. longum* subsp *infantis, Bifidobacterium longum* subspecies *infantis; B. longum* subsp *longum, Bifidobacterium longum* subspecies *longum*; DNA, deoxyribonucleic acid; HEI, HIV-exposed infected; HEU, HIV-exposed uninfected; HUU, HIV-uninfected; MWH, mothers with HIV; rRNA, ribosomal RNA; SCFAs, short-chain fatty acids; PCR, polymerase chain reaction; *S. aureus, Staphylococcus aureus; S. homonis, Staphylococcus homonis, S. paranguis, Staphylococcus paranguis*; WGS, whole genome sequencing; HIV, human immunodeficiency virus; V3, hypervariable region 3; V4, hypervariable region 4.

### Antiretroviral drugs used in sub-Saharan Africa

Recommendations of ARV drugs in many SSA countries align with WHO guidelines.^29^ Though the region is multi-contextual, SSA constituted 60% of global HIV infections in 2020.^39^ Over two million children are thought to be living with HIV worldwide, of whom over 80% live in the SSA region.^31^ No wonder, over one million infants from SSA are born to MWH and are potentially exposed to ARV drugs during antepartum, intrapartum and postpartum stages.^32^ There is, however scarcity of published studies on the gut microbiome of infants exposed to HIV and ARV drugs from SSA. There are even fewer studies from regions outside SSA, though findings are somewhat similar.^40^

### Original research focusing on the gut microbiota of sub-Saharan African infants exposed to antepartum, intrapartum and postpartum antiretroviral drugs

#### Cameroon findings

In Cameroon, in addition to NVP and azidothymidine (AZT) also known as ZDV, 3TC, abacavir (ABC) and lopinavir and ritonavir (LPV and r) are given to infants infected with HIV.^29^ One Cameroonian study found that children on ritonavir-boosted protease inhibitor (PI and r)-based ART had lower alpha diversity and altered gut microbiota composition compared to children on NVP and those uninfected with HIV.^29^ The gut microbiome of children born with HIV was largely characterised by a depletion of *Segatella* and *Clostridia*.^29^ Since the CWH were receiving both ART and prophylactic co-trimoxazole, it’s challenging to determine the individual impact of each medication on gut microbial diversity.^29^ A decrease in *Segatella* may indicate reduced fibre fermentation capacity, which can result in poorer gut health and lower production of short-chain fatty acids (SCFAs). The SCFAs – acetate, propionate and butyrate – are key mediators of gut microbiome benefits.^41^ They maintain gut integrity, support immune function and regulate host metabolism.^41^ While increased SCFA production is associated with health benefits, most evidence comes from animal studies, with limited data from well-controlled human trials.^39^ In addition, a reduction in *Clostridia* may suggest an imbalance in the microbiome, potentially increasing susceptibility to infections or inflammatory conditions.^29^

In another Cameroon study conducted among 80 infants (41%) were HEI, 15 (18.8%) were HEU and 32 (40%) were HUU.^31^ Of the 33 HEI infants, 32 (97%) were on ARV treatment. A statistically significant difference was observed between the number of HEI versus HEU infants harbouring *Clostridium, Enterococcus, Klebsiella, Shigella, Staphylococcus aureus (S. aureus)* and *Streptococcus*.^31^ The gut bacteria of HEI infants showed an imbalance, with a higher presence of opportunistic and pathogenic bacteria compared to HEU infants. The most common bacteria found in HEI infants were *Lactobacillus, Streptococcus* and *Bifidobacterium*. In HUU infants, *E. coli, Lactobacillus* and *Bifidobacterium* were most common. Some bacteria were notably absent in each group, for example, *Actinomycetes*, and *Enterobacter* were missing in HEI infants, as were *Acinetobacter, Proteus* and *S. aureus* in HUU infants.^31^

#### Mozambican findings

One study from Mozambique reported on the faecal microbiota of apparently healthy African infants from a community with a high HIV prevalence.^32^ A total of 120 faecal samples were collected. Four mothers reported taking medications at the time of the study, including amoxicillin, paracetamol, co-trimoxazole and antihypertensive drugs. Approximately 38% of infants aged < 6 months were exclusively breastfed.^32^ Twenty-nine MWH had detectable virus. *Streptococci, Enterococci* and *Bifidobacteria* were detected in all samples analysed, while *Staphylococcus* was the bacterial group least detected. The study found commonly detected bacteria such as *Streptococcus, Enterococcus* and *Bifidobacterium*, and the least detected *Staphylococcus*. Bacterial counts changed with infant age. *Bifidobacterium, Bacteroides, Enterococcus, Clostridium leptum* and *Clostridium coccoides* counts increased with infant’s age^32^ whereas *Staphylococcus aureus* and *Streptococcus epidermidis* counts were significantly reduced in older infants.^32^

#### Nigerian findings

A 2022 study in Nigeria showed marked differences in nutritional and microbiota diversity between HEU and HUU infants.^1^ In the study, HEU infants consistently exhibited lower weight-for-age z-score (WAZ) compared to HUU infants across all measured time points.^1^ At 6 months postpartum, a significant observation was made regarding the relative abundance of *Bifidobacterium* in HEU infants,^1^ which was found to be lower than that in HUU infants.^1^ It was noted that NVP and 3TC were present in the breast milk of MWH.^1^ *Bifidobacterium* provides numerous health benefits, including supporting digestive health, enhancing immune function, producing essential nutrients, preventing chronic diseases, aiding infant development and influencing mental health.^40,42,43,44,45^

Another study compared the gut microbiota of HEU and HUU infants born to 278 mothers with or without HIV, from Nigeria and South Africa.^33^ All 212 MWH received ART according to local guidelines, and their infants received NVP post-exposure prophylaxis after birth.^33^ The gut microbiota of Nigerian infants was primarily dominated by *Bifidobacterium longum* subspecies *infantis* and the Firmicutes, including both *Staphylococcus* species^33^ (e.g. *S. haemolyticus, S. saprophyticus*) and *Enterococcus* species (e.g. *E. faecalis, E. faecium*).^33^ In South African infants, certain bacterial taxa were linked to HIV exposure. Notably, several Enterococcus species were more abundant in HEU infants compared to HUU infants at week 1.^45^ In South African infants, the gut microbiota typically consisted of *Bifidobacterium* species^45^ (*B. longum, B. catenulatum, B. breve*), Firmicutes (*Streptococcus* species: *S. salivarius, S. caprae, S. lutetiensis*) and Pseudomonadota (mainly *Escherichia coli*).^33^ Pathobionts were more abundant in HEU infants during the first 15 weeks of life in the South African cohort.^45^ In contrast, no bacterial taxa were differentially abundant by HIV exposure in the Nigerian cohort.^33^

#### South African findings

One other South Africa-based study focused on the oral microbiome of school-aged CWH on ART.^11^ A few other studies in South Africa indicated great discrepancies in the interplay of the gut microbiota in HE infants.^34^

Another South African study analysing meconium and tracking infant faecal bacteria over time (birth to 20-28 weeks) found that in HIV-unexposed infants, *Pseudomonadota* (formerly *Proteobacteria)* decreased with age, and Clostridia increased with age.^11^ The impact of infant age on faecal bacterial profiles was less pronounced in HE infants.^11^ Higher levels of *Pseudomonadota* during infancy may correlate with slower growth trajectories and could predispose infants to metabolic disorders, such as obesity and insulin resistance later.^46^ *Clostridia* are known to be involved in the fermentation of dietary fibres and the production of SCFAs, such as butyrate.^47^ Short-chain fatty acids benefit gut health by providing energy to colon cells, maintaining intestinal barrier function and reducing inflammation.^47^ An increase in *Clostridia* may lead to higher SCFA levels, contributing to improved gut health and potentially reducing the risk of gastro-intestinal diseases.^48^ Therefore, the effect of infant age on stool microbiota among HE infants was less pronounced. This may imply that exposure to HIV or ART alters the typical developmental trajectory of the gut microbiome, leading to different patterns in bacterial composition as these infants age, which has implications for growth, metabolic disorders and gut health.

A South African study found that at 6 weeks, HEU infants had a higher abundance of *Firmicutes, Carnobacteriaceae* and *Enterococcaceae* compared to HUU infants.^11^ Conversely, it showed a lower abundance of the *Coriobacteriaceae*, and *Bacteroidetes* families, and a reduced abundance of the *Pseudomonadota* phylum,^11^ suggesting that the gut microbiome of HEU infants may be adapting differently compared to their HUU counterparts. *Firmicutes* are often associated with beneficial metabolic functions, including energy harvest from dietary fibres.^39,49,50^ The lower abundance of *Coriobacteriaceae* and *Bacteroidetes* in HEU infants could imply a potential disruption in the diversity and functionality of the gut microbiome.^51,52^ *Bacteroidetes* are generally linked to a healthy gut environment and play roles in breaking down complex carbohydrates.^51,52^

A 2016 South African study used shotgun metagenomic sequencing to analyse the gut microbiota of 4-week-old infants, comparing HUU neonates and HEU neonates before starting co-trimoxazole prophylaxis in the HEU group.^34^ There was no difference in microbial alpha diversity between HUU and HEU neonates.^34^ Forty-three bacterial taxa were found to be significantly differentially abundant between HUU and HEU.^34^ Compared to HUU neonates, HEU neonates had higher levels of pathobionts^34^ (*Shigella flexneri, Shigella boydii* and *Klebsiella pneumonia*) and other taxa, that is, *Blautia liquoris, Coprococcus eutactus* and *Roseburia hominis*.^34^ In contrast, HUU neonates had higher levels of *Bifidobacterium breve, Actinomyces fecalis, Eggertella lenta* and *Phascolarctobacterium* spp.^34^

Random forest analysis identified key metabolic pathways that distinguished HEU infants, with elevated levels of glycogen degradation, L-arginine degradation, L-methionine and L-rhamnose biosynthesis.^34^ Other pathways associated with HEU status were inosine-5-phosphate biosynthesis, lactose and galactose degradation and mixed acid fermentation.^34^ In HUU infants, the following pathways were elevated: pentose phosphate pathway, phospholipid biosynthesis and nitrate reduction pathway.^34^ The study found no significant impact of *in utero* HIV exposure on microbial diversity in infants at 4 weeks of age.^34^ However, after birth, HIV exposure alters gut bacterial microbiota composition, with HEU neonates displaying a higher abundance of pathobionts compared to HUU neonates.^34^ Human immunodeficiency virus exposure also seems to impact the functional metagenome in these neonates.^34^

A 2020 study compared the innate response and the stool microbiome of 2-year-old HEU and HUU children from Belgium, Canada and South Africa.^35^ The study reported no universal immune or microbiome signature underlying differences between HEU versus HUU that was applicable to all children.^35^ However, population-specific differences in stool microbiomes were readily detected. South African HEU and HUU children differed for WAZ,^35^ and differences in height-for-age z-scores (HAZ) approached significance.^35^ In this study, MWH formula-fed their infants to prevent transmission, while HUU infants were breastfed.^35^ According to the study findings, HIV exposure had a minimal impact on gut microbiota in South African children. However, differential abundance was observed for *Rikenellaceae*, more abundant in HEU and *Succinivibrio*, more abundant in HUU children.^35^ The authors hypothesise that breast versus formula feeding may have contributed to microbiome differences between HEU and HUU children in their study.^35^

A 2022 poster presentation reported findings from South Africa.^36^ The South African study compared 79 infants with intrauterine HIV infection (49% female), starting ART within 48 h or around 6 days of birth and 71 uninfected infants (52% female), born to MWH, receiving standard prophylaxis.^36^ The study collected rectal swabs at 4 weeks, 12 weeks and 24 weeks, analysing the microbiome through 16S ribosomal ribonucleic acid (rRNA) gene sequencing (V4 region).^36^ Human immunodeficiency virus infection was linked to notable changes in gut microbiota, with increased abundance of *Bacteroides, Fusobacterium* and *Finegoldia*, after accounting for other influencing factors.^36^ Other key factors influencing infant microbiome development were HIV status, age at sampling, mode of delivery, breastfeeding and sex.^35^ The study found that breastfeeding, combined with early ART, helped maintain a *Bifidobacterium*-rich microbiota profile in infants with HIV.^36^

#### Zimbabwe findings

Human immunodeficiency virus-exposed infants in Zimbabwe are exposed to one or more of the following ARV drugs: DTG, NVP, FTC, 3TC, TDF and AZT. A study described the composition of gut microbiota of children and adolescents, aged 6 to 16 years,^17^ with perinatal HIV infection taking ART in Zimbabwe.^17^ The study reported findings from 177 HEI and 106 HUU participants.^17^

Key findings showed that 89% of HEIs were on co-trimoxazole prophylaxis and that recent antibiotic use did not alter the impact of HIV status on species richness.^17^ Compared to HUU, HEI had a lower abundance of Epsilonbacteraeota and Bacteroidetes (phylum level)^17^ and enrichment of *Corynebacterium, Lawsonella, Collinsella* (Actinomycetota phylum), *Finegoldia, Anaerococcus, Erysipelotrichaceae, Lachnoclostridium* (Firmicutes phylum).^17^ Human immunodeficiency virus-unexposed uninfected participants, compared to HEI were enriched in *Campylobacter*,^17^ phylum Epsilonbacteraeota; *Porphyromonas* and *Prevotella*, phylum Bacteroidetes.^17^ There was also enrichment in the *Fastidiosipila, Fournierella, Eubacterium coprostanoligenes*_group, *Ruminococcaceae, Coprococcus* and *Murdochiella* (Firmicutes).^17^ Long-term ART (≥ 10 years) was associated with a more diverse gut microbiota in children, similar to that of HUU children, potentially minimising microbiota differences between HEI and HUU children.^17^

Another Zimbabwean study found no significant differences in gut microbiota diversity between HEU and HUU infants, as measured by alpha diversity indices (Shannon and Simpson) and stool microbial composition.^37^

A 2024 study reported on the infant gut microbiota members isolated from the stool of 6-week-old neonates from Zimbabwe.^53^ The study provided preliminary insights into the genomic makeup of specific strains of *Bifidobacterium* and *Enterococcus* recovered from an infant cohort from Zimbabwe,^53^ and explored some of the properties that allow these organisms to thrive in the infant gut environment.^53^ The final dataset comprised 6 type strain genomes representative of infant-associated *Bifidobacterium* and *Enterococcus* species^53^ – *Bifidobacterium longum* subsp. *infantis, Bifidobacterium longum* subsp. *longum, Bifidobacterium breve, Bifidobacterium pseudocatenulatum, Enterococcus faecium* and *Enterococcus faecalis*.^53^

### Common Metagenomics techniques in sub-Saharan African studies

Several metagenomics techniques can be employed to investigate the gut microbiota of HE infants in Africa.^14^ These include the 16S rRNA gene sequencing, shotgun sequencing and whole genome sequencing of cultured isolates.^6^ Most of the sub-Saharan African studies utilised 16S rRNA amplification, which has limited ability to discriminate species or strain level information, nor to predict microbial function.^1^ The reason for selecting the 16S rRNA gene amplicon sequencing method could be mainly because of its low cost, although none of the authors specified this. Several Sub-Saharan African studies assessed the gut microbiota in infants with variable results, likely because of different HIV exposure status,^1^ antibiotic usage, different age groups, a range of sample sizes, different modes of feeding (exclusive breastfeeding vs. formula)^1^ or cohorts from different geographical areas, all of which may impact results.^1^

### Overall summary of findings

There is still controversy amid the scarcity of data in the context of HIV or ART from the sub-region: 12 articles studied the effects of HIV and different ARV prophylaxis drugs on the gut microbiome of less than 1800 infants of varying age groups, from SSA.^12^ The 12 studies reported here focused mainly on breastfed infants (with some exclusively breastfed and some on mixed feeds). A few (less than 50%) of the studies followed up infants from birth to over 12 months. Using rRNA gene amplicon sequencing methods, the studies seem to disagree about whether there are differences in alpha and beta diversity of gut microbiota between the HE and HU infants from SSA. Those studies that delved deeper using shotgun metagenomics report differences in certain phyla, genus and even strains of important bacteria such as *Bifidobacterium*. The heterogeneity of age groups, feeding methods and maternal HIV status makes it difficult to make definitive conclusions. What we found clear is that more in-depth studies, using shotgun sequencing, whole genome sequencing in larger cohorts, followed by randomised clinical trials, are more appropriate in this context.

### Limitations and strengths of the study

Though this multi-context study has limitations inherent in such studies, this scoping review facilitates understanding of how the impact of HIV and/or ART on infant microbiota differ across different Sub-Saharan African contexts.^54^ The scoping review also helped us identify gaps in several contexts. Its findings will benefit from further preliminary studies or primary research in local contexts. Single-context research in different Sub-Saharan African countries will be necessary to corroborate findings from this multi-context review. Most studies reported in this scoping review used 16S rRNA gene amplification methods, despite their limitations in species or strain level resolution and prediction of microbial function. The reasons for the choice of methods are not specified, though low cost could be a contributing factor. The low resolution of the bacterial phylum and genus may contribute to the variability in findings.

## Implications and recommendations

Though findings are still controversial, the reviewed studies demonstrate that ART exposure can significantly alter the gut microbiota composition and diversity in HE infants and infants with HIV.^35^ The reported alterations, including decreased bacterial diversity and increased *Proteobacteria*, particularly *Enterobacteriaceae* abundance, may have implications for immune system development and long-term health outcomes.^29,47,48,52,55^ The microbial imbalance is likely shaped by multiple early-life exposures, including maternal HIV infection, ART, altered breastfeeding practices, increased antibiotic use and heightened perinatal immune activation. From a pathophysiological standpoint, reduced bacterial diversity limits the spectrum of microbial-associated molecular patterns available to prime and regulate the infant’s developing immune system. This impairs the balanced maturation of gut-associated lymphoid tissue, skewing immune development toward a pro-inflammatory state and reducing the induction of immune tolerance via regulatory T cells (Tregs). Biochemically, the over-representation of *Pseudomonadota* (formerly *Proteobacteria*) contributes to a more inflammatory gut environment. Many *Pseudomonadota*, such as *Escherichia coli* and other *Enterobacteriaceae*, possess highly immunostimulatory lipopolysaccharide structures that potently activate Toll-like receptor 4 (TLR4) on intestinal epithelial cells^46^ and innate immune cells.^29,38,51^ This results in upregulation of pro-inflammatory cytokines such as interleukin 6 (IL-6), tumor necrosis factor (TNF)-α and interleukin-1 beta (IL-1β),^41^ perpetuating mucosal inflammation and compromising epithelial barrier integrity. In addition, HIV-associated immune activation, in infants with HIV, may enhance gut epithelial oxygenation and nitrate production, favouring *Pseudomonadota* expansion through anaerobic-to-aerobic metabolic shifts.^47,48,52^ According to some of the studies in this scoping review, the gut environment of HE infants is likely to experience a decrease in obligate anaerobes, such as *Bifidobacterium* and *Faecalibacterium*, which are critical for producing SCFAs such as butyrate and acetate. Short-chain fatty acids are key epigenetic and immunomodulatory metabolites that promote Treg differentiation, support intestinal barrier function and suppress excessive inflammatory signalling.^29,47,48,52,55^ Their depletion in infants on ART impairs these protective mechanisms and may predispose them to long-term health consequences, including increased susceptibility to infections, allergic disorders, growth faltering and potentially metabolic or autoimmune diseases later in life.^29,47,48,52,55^

Thus, the combination of microbial immaturity, low diversity and *Pseudomonadota*-dominant microbial disturbances in infants on ART represents a biologically significant disruption of early-life gut-immune cross-talk with potential lifelong health implications. These observations must be explored in well-defined and comprehensive cohort studies with comparative groups of similar age and feeding characteristics.^29,47,48,52,55^

## Conclusion

There is a high burden of HIV in women of childbearing age, hence it is crucial to carry out studies focusing on the composition of both gut bacteria and non-bacterial components of the microbiota, such as fungi, viruses or bacteriophages. An interrogation of whether ARV drugs have a more pronounced impact on non-bacterial microbiota is worth exploring. Future studies are also required to screen antibiotics for their impact on the various gut bacteria in the Sub-Saharan African setting. Such research will inform the design of next-generation interventions, including prebiotics and probiotics tailored for infants from SSA.

A clinical trial in adults living with HIV, reported that the *Bacteroidales : Clostridiales* ratio was inversely correlated with HIV reservoir size and viral control post-analytical ART interruption,^20^ highlighting the potential contribution of the microbiome of an individual on their HIV persistence status.^20^ Whether this HIV-induced modulation of the gut microbiome creates an immune environment that provides a competitive advantage for reduction of a stable HIV reservoir needs further investigation, in light of HIV cure efforts.^38^

## Appendix 1

BOX 1 Supplementary document showing some of the search strategies.

**Table:**

**A1.1 Cochrane Library Search Terms for Title/Abstract/Keywords** Infant or neonate or children or child or baby or babies or newborn or new-born or ‘new born’ or ‘early childhood’ AND HIV or AIDS or ‘Human immune deficiency virus’ or ‘Acquired immunodeficiency syndrome’ or ‘maternal syndrome’ AND nevirapine or viramune or nevimune or ‘non-nucleoside reverse transcriptase inhibitors’ or NNRTI or ‘antiretroviral therapy’ or ART or ‘ARV prophylaxis’ or ‘sub-Saharan Africa’ or SSA	
**A1.2.1 PUBMED Search Terms for Title/Abstract** ((((((((((‘Sub-Saharan Africa’[Title/Abstract]) OR SSA[Title/Abstract]) OR Africa[Title/Abstract]) OR (((Infant[Title/Abstract]) OR (Neonate[Title/Abstract])) OR (children[Title/Abstract])) OR (baby[Title/Abstract])) OR (babies[Title/Abstract])) OR (newborn[Title/Abstract])) OR (new-born[Title/Abstract])) OR (‘new born’[Title/Abstract])) OR (‘early childhood’[Title/Abstract])) AND (((((((HIV[Title/Abstract]) OR (AIDs[Title/Abstract])) OR (Human immune deficiency virus[Title/Abstract])) OR (Human immunodeficiency virus[Title/Abstract])) OR (Acquired immune deficiency syndrome[Title/Abstract])) OR (‘Acquired immunodeficiency syndrome’[Title/Abstract])) OR (maternal virus[Title/Abstract]))) AND (((((nevirapine[Title/Abstract]) OR (ART[Title/Abstract])) OR (‘Antiretroviral Therapy’[Title/Abstract])) OR (‘Antiretroviral prophylaxis’[Title/Abstract])) OR (NVP[Title/Abstract])) OR (‘Non-nucleoside reverse transcriptase inhibitor’[Title/Abstract])) OR (NNRTI[Title/Abstract]))))	
**A1.2.2 Alternative PUBMED Search Terms for Title/Abstract/Keywords** ((((SSA[Title/Abstract]) OR ((‘Sub-Saharan Africa’[Title/Abstract]) AND (((nevirapine[Title/Abstract]) OR (viramune[Title/Abstract])) OR (NVP[Title/Abstract])) OR (‘Non-nucleoside reverse transcriptase inhibitor’[Title/Abstract])) OR (NNRTI[Title/Abstract]) OR (ART[Title/Abstract]) OR ‘Aniretroviral therapy’[Title/Abstract]))))	
**A1.3 Ovid Search Terms for Abstract** ‘acquired immune deficiency syndrome’, immunodeficiency, aids, babies, baby, children, ‘early childhood’, hiv, human virus, infant, neonate, nevimune, nevirapine, ‘new born’, new-born, newborn, nnrti, ‘non-nucleoside reverse transcriptase inhibitor’, nvp, viramune, ‘prophylaxis ARV’, ART, ‘antiretroviral therapy’, SSA, ‘sub-Saharan Africa’	
**A1.4 ProQuest Theses and Dissertations Global Search** Combined Search 2, 4, 6 and 8 below:	
**A1.4.1**	**Search 2 terms:** abstract(Infant) OR abstract(neonate) OR abstract(baby) OR abstract(babies) OR abstract(newborn) OR abstract(new born) OR abstract(early childhood)
**A1.4.2**	**Search 4 terms:** abstract(HIV) OR abstract(AIDs) OR abstract(‘Human immune deficiency virus’) OR abstract(‘human immunodeficiency virus’) OR abstract(‘acquired immunodeficiency syndrome’) OR abstract(‘acquired immune deficiency syndrome’) OR abstract(‘maternal virus’)
**A1.4.3**	**Search 6 terms:** abstract(nevirapine) OR abstract(viramune) OR abstract(nevimune) OR abstract(NVP) OR abstract(‘non-nucleoside reverse transcription inhibitors’) OR abstract(NNRTI) OR abstract (Antiretroviral)
**A1.4.3.4**	**Search 8 terms:** abstract(‘Sub-Saharan Africa’) OR abstract(SSA) OR abstract(Africa)
